# Pediatric Lupus Retinopathy: A Rare Manifestation of a Grave Systemic Disorder

**DOI:** 10.7759/cureus.46616

**Published:** 2023-10-07

**Authors:** Shweta Parakh, Vaibhav Bhatt, Shrutanjoy Das, Abhijaat Chaturvedi, Gaurav Luthra, Deeksha Katoch, Prabal Barman, Ankur K Jindal, Saurabh Luthra

**Affiliations:** 1 Ophthalmology, Drishti Eye Institute, Dehradun, IND; 2 Ophthalmology, Postgraduate Institute of Medical Education and Research, Chandigarh, IND; 3 Pediatric Rheumatology, Postgraduate Institute of Medical Education and Research, Chandigarh, IND

**Keywords:** antinuclear antibody (ana), anti-extractable nuclear antigen (ena) antibodies, vasculopathy, immunosuppression, pediatric lupus retinopathy, pediatric systemic lupus erythematosus (psle)

## Abstract

We describe a rare case of pediatric systemic lupus erythematosus (pSLE) and its successful management. A nine-year-old female presented with bilateral diminution of vision, fever, and rash in the malar region, chest, abdomen, back, and arms for three months. Clinical examination and multimodal imaging revealed bilateral extensive retinal vasculitis with macular edema. Laboratory investigations revealed anemia, leucopenia, positive serum antinuclear antibody (ANA), and anti-extractable nuclear antigen (ENA) antibodies. A diagnosis of pediatric lupus retinopathy was made. Ocular and systemic manifestations responded well to intense systemic immunosuppression (pulse intravenous {IV} methylprednisolone, oral prednisolone and hydroxychloroquine {HCQ}, six cycles of IV cyclophosphamide, and oral azathioprine) along with topical steroids and laser photocoagulation, over the next 10 months.

Though ocular manifestations are not a part of the diagnostic criteria for SLE, they may be markers of active systemic disease. Ophthalmologists and rheumatologists must treat this complex disease in tandem in order to provide optimum patient care.

## Introduction

Systemic lupus erythematosus (SLE) is a complex chronic multisystem autoimmune inflammatory disorder. Pediatric systemic lupus erythematosus (pSLE) includes, by definition, a subset of patients with disease onset before 18 years of age [1]. With the mean age at the onset of SLE symptoms characteristically being between 20 and 40 years, pSLE represents approximately 10% of SLE cases [2]. Falling under the criteria of a rare disease in Europe [3], pSLE has an incidence of 0.3-0.9 per 100,000 children years and a prevalence of 1.89-25.7 per 100000 children worldwide [4-6] including in Europe [7-11].

Keratoconjunctivitis sicca is the most common ocular manifestation, whereas retinal and choroidal involvements are most associated with sight-threatening disease [12,13]. The latter is usually associated with systemic features, thus aiding early diagnosis and prompt treatment [14-16].

We report a rare case of pSLE in a nine-year-old female who presented with bilateral lupus vasculitic retinopathy and dermatological signs that led to a prompt diagnosis and successful management by an expert team of pediatric rheumatologists and retina specialists.

## Case presentation

**Figure 1:**
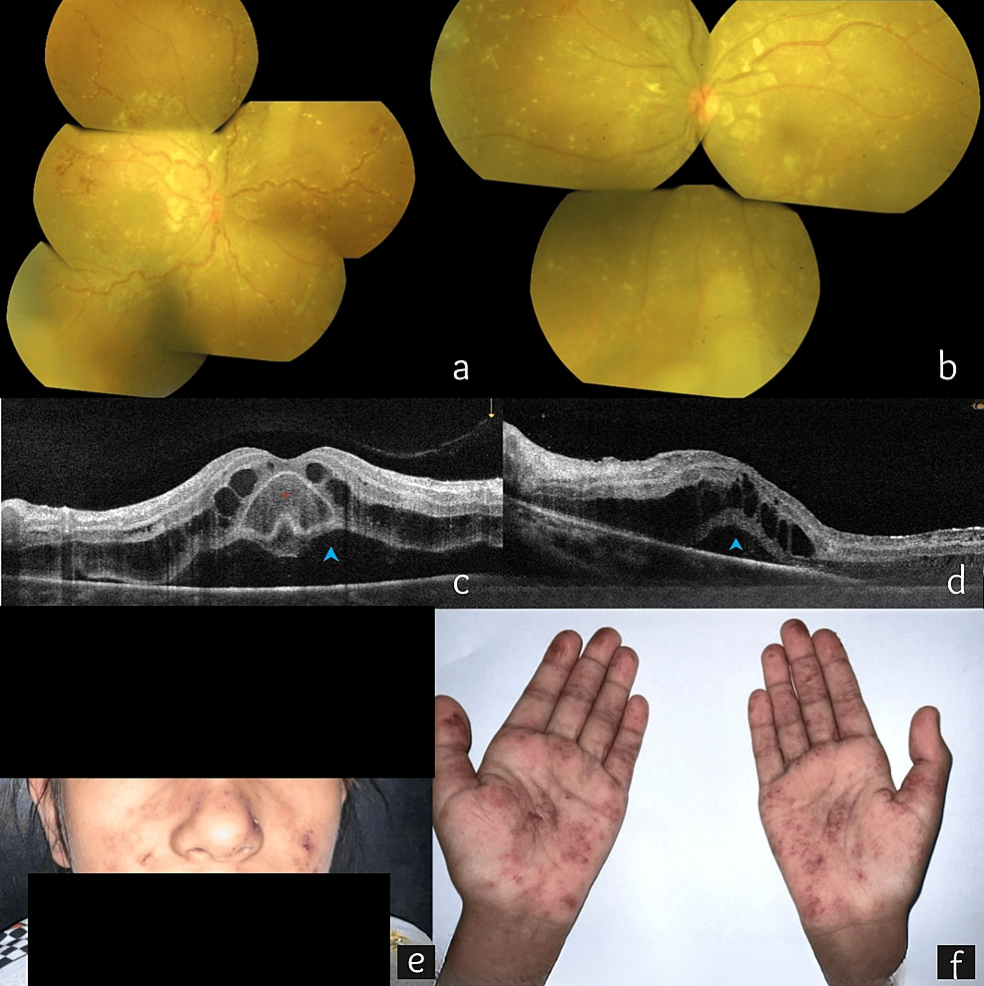
At the first visit. (a and b) Color fundus image (montage) at initial visit showing bilateral disc hyperemia, cystoid macular edema, peripapillary areas of greying, and extensive perivascular sheathing in the mid-periphery along with perivascular subretinal infiltration. (c and d) Spectral domain optical coherence tomography (SD-OCT) showing bilateral cystoid macular edema, neurosensory detachment (blue arrowhead), and inner retinal hyperreflectivity. (c) Prominent bacillary layer detachment (red star) was seen in the right eye. (e and f) A diffuse erythematous rash can be seen over the malar region and palms in the acute phase.

A nine-year-old female presented to us with bilateral gradual diminution of vision, pain, and redness for 15 days. The best-corrected visual (BCVA) acuity was 6/60 in both eyes (OU). Intraocular pressure (IOP) was normal. The pupils were equal, round, and reactive to light and accommodation. The anterior chamber showed trace cells and mild flare in both eyes. Fundus examination showed bilateral disc hyperemia, cystoid macular edema (CME), peripapillary areas of greying suggestive of choroidal ischemia, and extensive perivascular sheathing in the mid-periphery along with perivascular subretinal infiltration (Figure 1a, 1b). Spectral domain optical coherence tomography (SD-OCT) (RTVue XR Avanti, Optovue Inc., Fremont, CA) showed bilateral neurosensory detachment (NSD) along with CME (central macular thickness measuring 891 μm in the right eye {OD} and 723 μm in the left eye {OS}) and inner retinal hyperreflectivity (Figure 1c, 1d). Prominent bacillary layer detachment (BALAD) was also noted OD (Figure 1c). Systemic evaluation was notable for irritability, fatigue, anorexia, fever, and arthralgia along with a diffuse erythematous nonpruritic rash over the malar region, chest, abdomen, back, palms, and extensor aspect of the arms for three months (Figure 1e, 1f). A provisional diagnosis of retinal vasculitis secondary to an unknown systemic illness was made. Topical prednisolone acetate 1% eye drops one hourly were started OU. The child was urgently referred to a pediatric rheumatologist for expert opinion.

Systemic investigations revealed microcytic hypochromic anemia with leukopenia (hemoglobin, 7.7 g/dL {normal range: 11.0-15.0 g/dL}; total leucocyte count, 2900/cu mm {normal range: 4500-14500/cu mm}). Differential leucocyte count showed absolute neutrophil count of 2030/cu mm (normal range: 1700-7500/cu mm) and absolute lymphocyte count of 812/cu mm (normal range: 1250-7000/cu mm). Erythrocyte sedimentation rate was markedly raised (99 mm in the first hour). The direct Coombs test was negative. Serum albumin was low at 2.2 g/dL (normal range: 3.2-4.5 g/dL). Serum creatinine was normal at 0.50 mg/dL (normal range: 0.30-0.70 mg/dL). Absolute cluster of differentiation (CD) 4 count was normal at 1383 cells/cu mm (normal range: 500-1500 cells/cu mm). Liver function tests were markedly elevated with alanine transaminase at 733 U/L (normal range: 5-25 U/L) and aspartate aminotransferase at 173 U/L (normal range: 10-50 U/L). Enzyme-linked immunosorbent assay (ELISA) for HIV tested negative. Serum antinuclear antibody (ANA) was positive (1:80 primary dilution, 4+ primary intensity on immunofluorescence on human epithelial type 2 {HEp-2} cells with speckled pattern, and 1:640 end-point titer). Anti-extractable nuclear antigen (ENA) antibodies such as anti-Smith, anti-Sjögren's-syndrome-related antigen A (SSA)/Ro-52, anti-Sjögren's-syndrome-related antigen B (SSB)/La, and anti-ribonucleoprotein (RNP) antibodies were strongly positive (+++). Serum immunoglobulins (Ig) were elevated: IgA was 347 mg/dL (normal range: 34-274 mg/dL); IgG was 2340 mg/dL (normal range: 462-1682 mg/dL). Serum complement C3 was low (52 mg/dL; normal range: 84-168 mg/dL). Rheumatoid factor and anti-double-stranded deoxyribonucleic acid (dsDNA) antibody tested negative. Chest X-ray was normal, interferon gamma release assay (IGRA) (QuantiFERON-TB Gold Test) was negative, and the Mantoux test was negative. Ultrasound of the abdomen showed mild hepatomegaly. A diagnosis of retinal vasculopathy secondary to pediatric systemic lupus erythematosus (pSLE) and juvenile dermatomyositis with mixed connective tissue disorder (overlap syndrome) was made by the team of pediatric rheumatologists. The patient was administered three doses of pulse intravenous (IV) methylprednisolone 125 mg, followed by oral prednisolone 10 mg twice daily and oral hydroxychloroquine (HCQ) 100 mg once daily. Intravenous cyclophosphamide 450 mg in 500 mL dextrose normal saline (DNS) (slow infusion over 3-4 hours) was also administered.

At three-week follow-up, the general condition of the child had improved. BCVA had improved to 6/18 OD and 6/36 OS. The anterior chamber was quiet. Fundus examination showed improvement with bilateral resolving CME and reduction in perivascular sheathing. SD-OCT showed OU reduction in NSD and CME and the resolution of BALAD in OD. Topical prednisolone acetate 1% eye drops were reduced to six times daily. Weekly tapering was done over the next six weeks.

**Figure 2:**
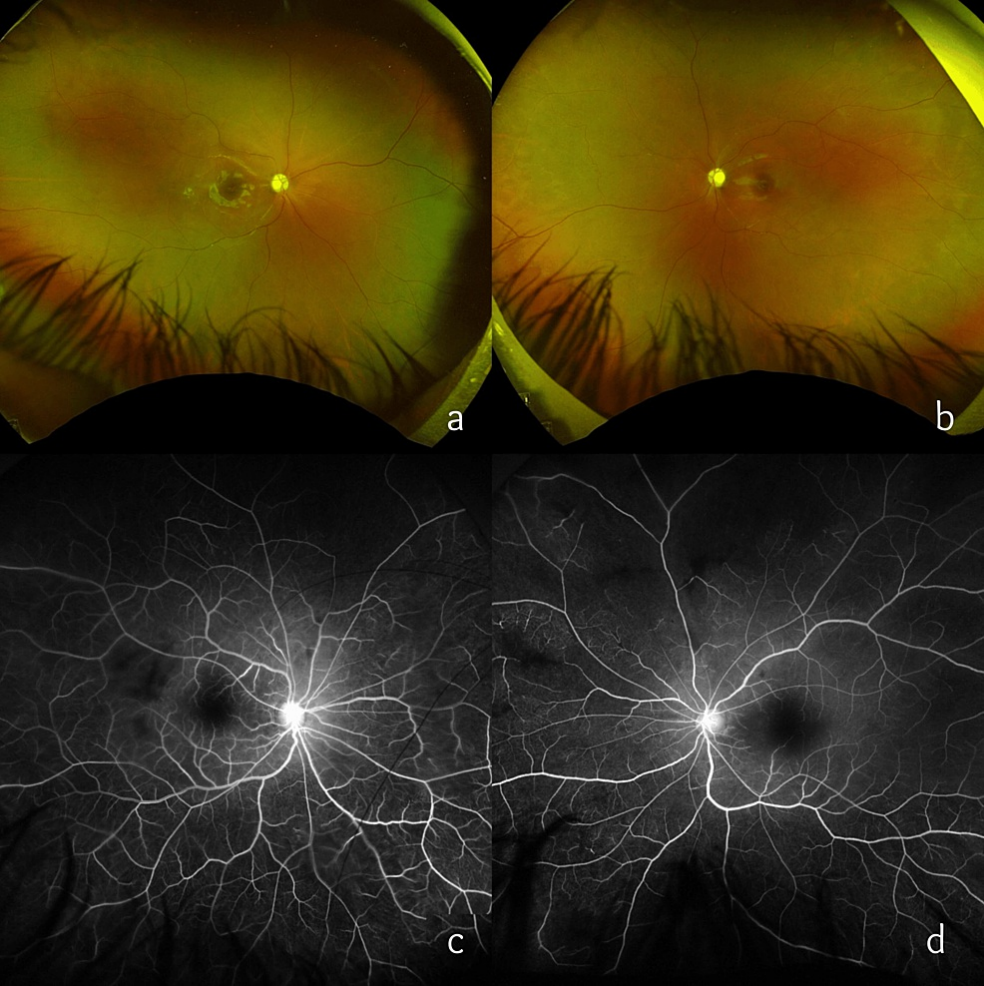
At the three-month follow-up visit after the initiation of systemic immunosuppressive therapy. (a and b) Ultra-widefield imaging showing bilateral disc pallor and the resolution of vasculitic signs noted at the first visit. (c and d) Ultra-widefield fluorescein angiography showing prominent bilateral disc staining and capillary non-perfusion areas in the periphery (OS > OD). OD, right eye; OS, left eye

Oral prednisolone was tapered gradually by 2.5 mg every four weeks over the next six months. Subsequently, she was maintained on oral prednisolone 2.5 mg once a day under the expert guidance and supervision of pediatric rheumatologists. The child also received six consecutive monthly doses of IV cyclophosphamide. Oral HCQ was continued at 100 mg once a day. Complete blood count, renal and liver function tests, and urine protein and blood coagulation profiles were monitored monthly and were all within normal limits. Oral azathioprine (75 mg and 50 mg on alternate days) was started three months after the initiation of IV cyclophosphamide. At the three-month follow-up visit, the child was symptomatically better. BCVA was 6/6 OD and 6/60 OS. Fundus examination revealed OU disc pallor with the resolution of CME and vasculitic signs (subretinal greying, cotton wool spots, perivascular sheathing, and vascular tortuosity) (Figure 2a, 2b). Fundus fluorescein angiography (FFA) showed peripheral capillary non-perfusion areas (OS > OD) (Figure 2c, 2d). One sitting of laser photocoagulation OS was done to the avascular peripheral retina.

**Figure 3:**
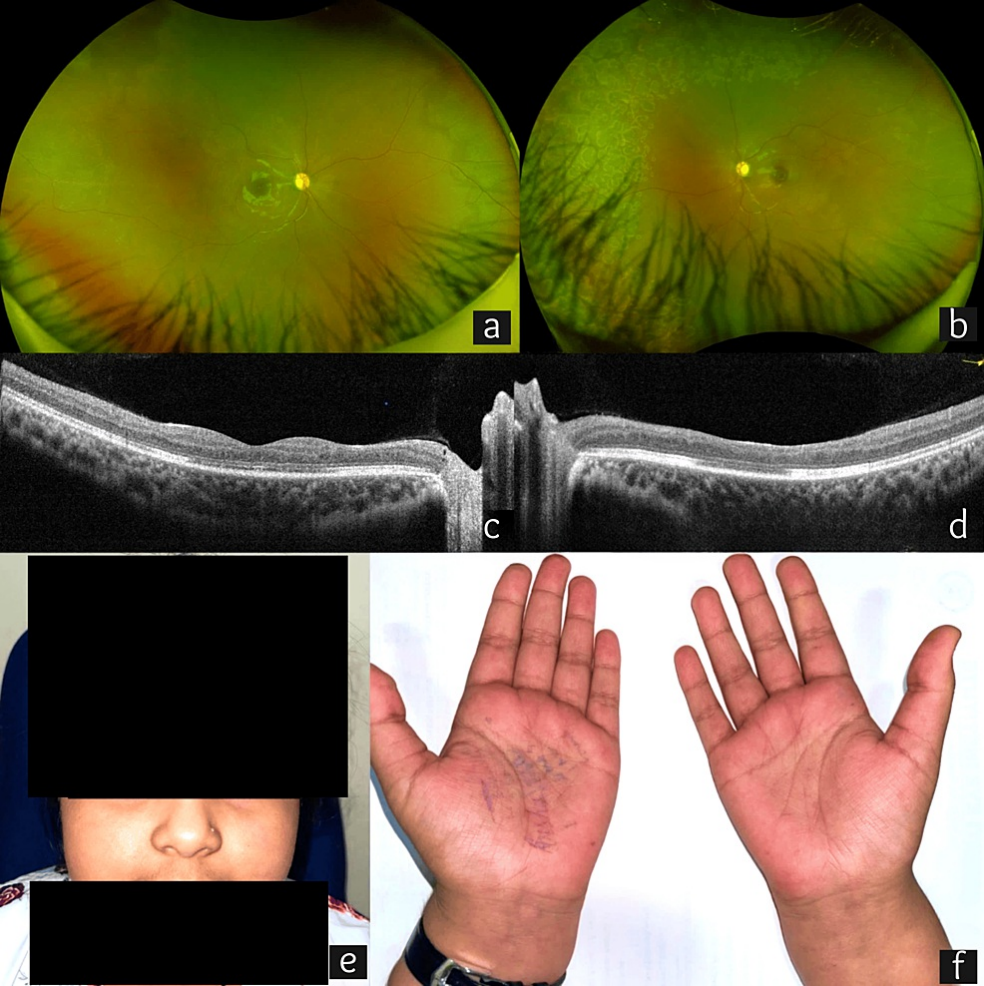
Imaging at 10-month follow-up visit depicting the resolution of active ocular and systemic disease. (a and b) Ultra-widefield fundus photo showing bilateral disc pallor. (b) The left eye (OS) shows well-lasered peripheral retina. (c and d) Spectral domain optical coherence tomography (SD-OCT) showing bilateral fine epiretinal membrane with gross macular thinning (OS > OD). (e and f) The resolution of the rash over the malar region and palms was noted initially. OD: right eye

At the latest follow-up visit at 10 months, the child showed marked ocular and systemic recovery. BCVA was stable at 6/6 OD and 6/60 OS. IOP was normal at 13 mm Hg OD and 10 mm Hg OS. The anterior chamber was quiet OU. Fundus examination showed disc pallor OU (Figure 3a, 3b). The peripheral retina was well lasered OS (Figure 3b). OCT showed a fine epiretinal membrane with gross macular thinning (OS > OD) (Figure 3c, 3d). OCT angiography showed flow void areas suggestive of macular ischemia (OS > OD). A review of systems showed a recovery of appetite, weight gain, and the resolution of arthralgia, fever, and rashes (Figure 3e, 3f). At the latest visit, systemic immunosuppressive therapy was tapered to oral azathioprine (50 mg once a week and 75 mg twice a week), oral HCQ 200 mg once a day, and oral prednisolone 2.5 mg once a day. Laboratory investigations were normal. The child continues to be under the close supervision of pediatric rheumatology and ophthalmology clinics.

## Discussion

Retinopathy is seen in 3%-29% of patients with SLE [17]. Pathologically, lupus retinopathy has two major mechanisms: direct by immune complex-mediated vasculitis [18] and indirect due to secondary hypertension from renal involvement. This manifests as retinal microangiopathy (cotton wool spots, microaneurysms, dot hemorrhages, and hard exudates), vasculitis (perivascular sheathing), and severe vaso-occlusion (central retinal artery or vein occlusion, neovascularization, vitreous hemorrhage, and tractional retinal detachment) [19]. The relatively innocuous microangiopathy features are mediated by immune complex deposition and inflammation, while the more ominous vaso-occlusive signs occur due to fibrinoid degeneration/necrosis without significant inflammation [12]. Central nervous system (CNS) vasculopathy shows a similar pathogenesis, thus explaining the strong correlation between CNS vasculitis and severe lupus vasculopathy [20,21].

In addition, lupus choroidopathy, a significant sight-threatening entity, can present as single or multiple areas of serous or exudative retinal detachment (36%), retinal pigment epithelium detachment (32%), or retinal pigment epitheliopathy (21%). Subretinal hypopigmented patches indicate choroidal ischemia and can be demonstrated on FFA as delayed choroidal filling, areas of choroidal non-perfusion, or multifocal areas of subretinal leakage with pooling corresponding to the areas of serous elevation and exudative retinal detachment [22]. Indocyanine green angiography (ICGA) can help to recognize the true extent of active choroidopathy not evident on clinical examination or FFA; it is characterized by focal transient early hypofluorescent areas and spots of choroidal hyperfluorescence in the intermediate to late phase [23]. OCT helps in monitoring active disease and the response of retinopathy and choroidopathy to systemic treatment by the qualitative evaluation of intraretinal fluid (IRF), subretinal fluid (SRF), and CME [24].

pSLE and adult-onset systemic lupus erythematosus (aSLE) differ regarding symptoms at onset, the pattern of organ involvement, and the severity of the disease [25]. pSLE is characterized by a less pronounced female/male ratio compared to the aSLE [2]; pSLE shows a higher frequency of renal, hematological, and neuropsychiatric involvement, while chronic cutaneous lupus is more commonly diagnosed in patients with aSLE [26]. Patients with pSLE also have higher disease activity compared to aSLE and commonly develop more organ damage, showing worse outcomes [27]. However, due to similarities in manifestation, the established American College of Rheumatology (ACR, 1997) classification criteria for aSLE [28] and the Systemic Lupus Erythematosus International Collaborating Clinics (SLICC, 2012) classification criteria [29] are widely used for pSLE [30]. Comparative studies between these criteria note that for pSLE, the SLICC criteria had greater sensitivity than the ACR criteria, albeit at the expense of losing some specificity [31,32].

Pediatric lupus retinopathy has been reported very infrequently in literature so far. The findings reported include combined vascular occlusion [33], vitreous hemorrhage with non-arteritic anterior ischemic optic neuropathy, and retinal vasculitis [34]. Our clinical findings were similar to those reported in the patient with retinal vasculitis. As accurately noted by Alhassan et al., there is a discrepancy regarding which ophthalmic findings (if any at all) are included in the disease activity scoring systems for SLE. Systemic Lupus Erythematosus Disease Activity Index 2000 (SLEDAI-2k) includes "visual disturbance," whereas the British Isles Lupus Assessment Group 2004 (BILAG-2004) specifies the part of the eye that is affected and the extent of involvement. Ocular manifestations are not a part of the European Consensus Lupus Activity Measurement (ECLAM) or the American College of Rheumatology (ACR) classification criteria [35].

The aim of the treatment of this rare and complex multisystem disorder is to induce and maintain the remission of the systemic illness and prevent relapses. Treatment options for ocular manifestations include systemic therapy (nonsteroidal anti-inflammatory drugs, hydroxychloroquine, systemic corticosteroids, immunosuppressive therapy, and biologics) and local therapy (laser photocoagulation, intravitreal anti-vascular endothelial growth factor {VEGF} injections, and vitrectomy). Providing a tailored approach according to the severity of the specific pathology is paramount and ideally involves a multipronged team effort including pediatric rheumatologists, nephrologists, dermatologists, and ophthalmologists. Commonly prescribed immunosuppressive drugs include azathioprine, cyclophosphamide, methotrexate, and mycophenolate mofetil. Targeted biological therapies in the form of monoclonal antibodies (e.g., rituximab, belimumab, epratuzumab, and sifalimumab) aimed against B and T lymphocytes, cytokines, and B cell-activating factors of the tumor necrosis factor (TNF) family (BAFF) are opted for in cases that do not respond to conventional immunosuppressive therapy [19]. Hydroxychloroquine is strongly recommended for the long term for all patients with SLE [36].

The role of panretinal photocoagulation is to suppress ischemic retinopathy and prevent neovascularization. Anti-VEGF drugs are an effective adjunctive modality for treating complications arising due to vaso-occlusion and vasculitis such as CME and retinal and choroidal neovascularization [37,38].

Our patient fulfilled the diagnostic criteria laid down by the European League Against Rheumatism (EULAR)/ACR 2019 classification criteria for SLE [39] with positive ANA as entry criterion and a high total score of 20 for the clinical and immunological criteria (fever, two points; leucopenia, three points; acute cutaneous lupus, six points; anti-Smith antibody positivity, six points; and low C3, three points). The ocular signs of retinal vasculopathy and choroidopathy in our case responded well to systemic immunosuppressive therapy.

## Conclusions

Though not a part of formal diagnostic criteria for SLE according to the American Rheumatology Association, retinal involvement can provide essential clues to disease activity due to the direct visualization of vascular phenomena. Therefore, both the ophthalmologist and the rheumatologist should be vigilant to the possible phenomenon of active lupus retinopathy portending systemic flare-ups. Ophthalmologists and rheumatologists should treat this complex disease and its wide-ranging manifestations in tandem in order to provide optimum patient care.
